# Polymeric β-cyclodextrin/alginate/*Colocasia esculenta* mucilage (β-CD/Alg/CEM) nanocomposites for the controlled delivery of 5-fluorouracil

**DOI:** 10.1039/d5ra07963b

**Published:** 2026-01-02

**Authors:** Maham Anoosh, Shazia Akram Ghumman, Huma Hameed, Shazia Noureen, Rizwana Kausar, Ali Irfan, Maged Ali A. Alrobesh, Mahwish Arshad, Pervaiz Akhtar Shah, Maria Rana, Yousef A. Bin Jardan

**Affiliations:** a College of Pharmacy, University of Sargodha Sargodha-40100 Pakistan anooshmaham173@gmail.com shazia.akram@uos.edu.pk; b Faculty of Pharmaceutical Sciences, University of Central Punjab Lahore 54000 Pakistan huma.hameed@ucp.edu.pk; c Institute of Chemistry, University of Sargodha Sargodha 40100 Pakistan shazianoureen11@gmail.com; d ILM College of Pharmaceutical Sciences Sargodha 40100 Pakistan rizvi_awan@yahoo.com; e The Green Institute of Chemical, Biomedical and Environmental Sciences (GICBES) Lahore-54000 Pakistan draliirfan.ceo@gicbes.com; f Department of Chemistry, Government College University Faisalabad Faisalabad-38000 Pakistan raialiirfan@gmail.com; g Pharmacy Services, King Saud University Medical City, King Saud University Saudi Arabia malropesh@ksu.edu.sa; h Roy & Diana Vagelos Laboratories, Department of Chemistry, University of Pennsylvania Philadelphia Pennsylvania 19104-6323 USA maarshad@sas.upenn.edu; i Department of Medicine, Perelman School of Medicine, University of Pennsylvania Philadelphia Pennsylvania 19104 USA; j University College of Pharmacy, University of the Punjab Lahore Pakistan pervaiz.pharmacy@pu.edu.pk; k Riphah Institute of Pharmaceutical Sciences, Riphah International University Lahore Campus Pakistan maria.rana@riphah.edu.pk; l Department of Pharmaceutics, College of Pharmacy, King Saud University Riyadh 11451 Saudi Arabia ybinjardan@ksu.edu.sa

## Abstract

5-Fluorouracil (5-FU) is a widely used cytotoxic chemotherapy drug in cancer management; its effectiveness in therapy is reduced due to a short half-life and toxic effects on healthy cells. This study aimed to overcome these limitations by preparing β-cyclodextrin/alginate/*Colocasia esculenta* mucilage (β-CD/Alg/CEM) nanocomposites containing 5-FU, designed for controlled release in various pH environments. Stable nanocomposites were successfully synthesized through the ionotropic gelation technique, achieving a drug content of 87.33 ± 1.75%, while the %age yield was found to be 79.06 ± 0.53%. Particle size analysis revealed a range of 80–100 nm with a polydispersity index (PDI) of 0.611. The zeta potential analysis showed that the nanocomposites possessed a surface charge of −27.1 mV. The nanocomposites displayed a porous and irregular morphology with a notably rough surface. In the acidic conditions of simulated gastric fluid (pH 1.2), the 5-FU release was markedly less than in the neutral conditions of simulated colorectal fluid (pH 7.4), indicating effective pH-sensitive release properties. The cytotoxic assay confirmed significant tumor–suppressive activity against MCF-7 and minimal effect on normal cells.

## Abstract 2

5-Fluorouracil (5-FU) is a widely used cytotoxic chemotherapy drug in cancer management; its effectiveness in therapy is reduced due to a short half-life and toxic effects on healthy cells.

## Graphical abstract

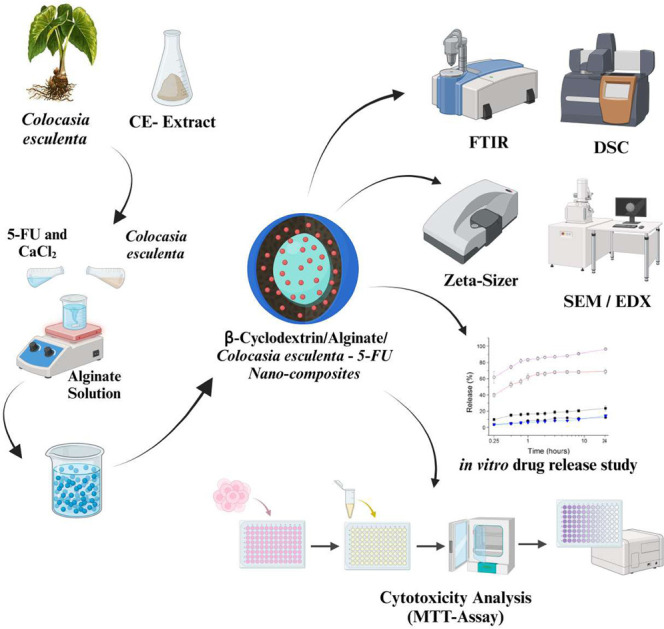

## 1. Introduction

5-Fluorouracil (5-FU) has played a major role in the battle against cancers, especially lung cancer,^1^ colorectal cancer,^2^ cervical cancer,^3^ and breast cancer for a long time.^4^ Although 5-FU is extensively metabolized by dihydropyridine dehydrogenase, exhibiting a short plasma half-life of 5–10 minutes.^5^ 5-FU also demonstrated broad-spectrum biological activity, targeting not only cancer cells but also normal cells, leading to cytotoxic effects visible in the alimentary tract and bone marrow.^6^ Over the past few years, experts have developed innovations to improve the drug's efficacy in fighting cancer and to better understand its therapeutic performance.^7^ Numerous investigations have explored synergistic treatment strategies involving co-therapies and advanced drug delivery platforms to enhance their efficacy in malignant tissues.^8^ Many polymer-based nanocomposites incorporating 5-FU have been created to restrict their dissolution and address such issues while maintaining excellent curative potential.^9^ These pharmaceutical carriers must have a significant ability to include drugs, a long half-life with exceptional stability in the circulatory system, targeted absorption at the desired location, and efficient drug dissolution in the right medium.^10^ Various advanced delivery systems, such as liposomal and lipid nanocarriers, have demonstrated improved tumor targeting and reduced systemic toxicity, while polymeric nanogels and pH-responsive carriers offered controlled and stimuli-triggered release in tumor-like acidic environments.^11–13^ Cyclodextrin-assisted formulations improved solubility and molecular protection of 5-FU through host–guest interactions, and alginate-based hydrogels, microspheres, and composite systems provided biocompatible matrices for sustained gastrointestinal and localized release. Although these strategies have shown promise, many still faced challenges such as low encapsulation efficiency, burst release, limited mucoadhesion, and inconsistent pH-dependent release profiles.

In recent times, numerous investigators have formed nanocomposites to allow substances to be released in a controlled manner.^14^ Folic acid–poly (lactic-*co*-glycolic acid) conjugates of 5-FU were formulated effectively and investigated against HT-29 tumor cells.^15^ SA/β-CD nanocomposite beads have been formulated for the treatment of colorectal cancer.^16^ SA/β-CD has also been utilized for the controlled release of 5-FU, demonstrating good potential as an anticancer drug delivery system.^17,18^ Few studies have been conducted on tripolymer systems, such as TG/β-CD/SA, which have been designed for the controlled release of aspirin.^19^ Cur/Cs-ALG-PVA nanocomposites have shown excellent potential for improved anticancer activity of curcumin.^20^ We have also utilized naturally occurring *Colocasia esculenta* mucilage (CEM), which is composed of a polysaccharide fraction consisting of mannose, glucose, xylose, galactose, and arabinose.^21^ The addition of *Colocasia esculenta* mucilage will enhance mucosal retention and enable controlled release, particularly for hydrophilic medications like 5-FU, by improving bio-adhesive properties and promoting gel-forming capability. CEM has a higher polysaccharide content than natural mucilages such as fenugreek, okra, basil seed, and cress seeds, which have been used as a pharmaceutical carrier. Due to the abundance of hydroxyl and carboxyl functional groups of CEM, it showed tremendous swelling, gel-forming characteristics, and strong mucoadhesive potential.^22^ Researchers have used *Colocasia esculenta* mucilage along with alginate to formulate microspheres containing pregabalin and oxcarbazepine,^23^ a polar extract of *Colocasia esculenta* has been used for the synthesis of silver nanoparticles.^24^

Although 5-fluorouracil (5-FU) is widely used in cancer therapy, its clinical success is restricted by rapid metabolism, short half-life, and nonspecific toxicity to healthy cells. To mitigate these limitations, the current study employed a β-cyclodextrin/alginate/*Colocasia esculenta* mucilage (β-CD/Alg/CEM) nanocomposite system, in which β-CD enhanced molecular encapsulation and protection of 5-FU, alginate provided structural integrity and controlled gel-based diffusion, and CEM contributed additional sustained-release and mucoadhesive characteristics. The synergistic features of this composite facilitated improved drug stability and controlled, pH-dependent release behavior.

## 2. Materials and methodology

5-Fluorouracil (received as a gift from Rotex Pharmaceuticals Pvt, Ltd), β-cyclodextrin (Sigma-Aldrich), sodium alginate: Mw 216, CAS-NO.: 9005-38-3 (Sigma-Aldrich), calcium chloride (Merck), and Deionized water served as the solvent for all solution preparations and mucilage extraction. Chemicals of analytical grade were used directly without any processing.

### 2.1. Isolation of *Colocasia esculenta* mucilage

*Colocasia esculenta* corms were purchased from a local market in Sargodha, Pakistan, in June 2023. The taro plant and corms were identified by taxonomist Prof. Amin Ullah Shah (Associate Professor from the University of Sargodha, Department of Botany), and a voucher specimen (No. UOS-PA-23-15) was deposited at the University of Sargodha's herbarium. The taro corms were completely cleansed with distilled water. Slices of equal length were macerated in water for 6 hours, bubbled at low temperature for 30 minutes, and pressed through muslin cloth. Ethanol was added to the filtrate to precipitate mucilage, which was dried at 40 °C, ground, sieved (#80), and stored in a sealed container.^24^

### 2.2. Preparation of 5-FU @ β-CD/Alg/CEM nanocomposites

**Fig. 1:**
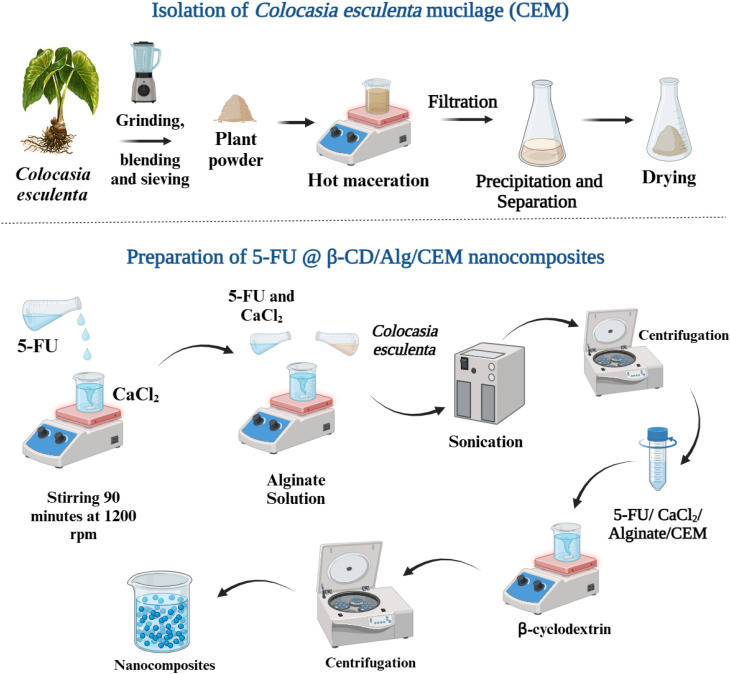
Schematic representation of isolation of *Colocasia esculenta* mucilage (CEM) and preparation of nanocomposites. Created in BioRender. Miller, S. (2025) https://BioRender.com/t36yt4m.

The 5-FU@β-CD/Alg/CEM nanocomposites were prepared using a modified ionotropic gelation method. Initially, an 8 mL 5-FU solution with (35 mg mL^−1^) was prepared, and then added dropwise to 5 mL of CaCl_2_ solution (6.5 mg mL^−1^, pH 6.5) and stirred for 90 minutes at 1200 rpm (25 ± 2 °C). The resulting suspension was combined with 6.8 mL of Alg solution (5 mg mL^−1^) and 6.8 mL of CEM solution (5 mg mL^−1^), agitated for 90 minutes, and subjected to ultrasonic radiation (40 kHz) for 60 minutes. After centrifuging and washing the 5-FU/CaCl_2_/Alg/CEM mixture with 3 × 10 mL of water, the β-cyclodextrin solution (10.7 mL, 1.6 mg mL^−1^) was added. After stirring and being left for 24 hours, the resulting product was centrifuged for 15 minutes at 3200 rpm and then rinsed with 3 × 10 mL of water. After 48 hours, the nanocomposites were produced by freeze-drying.^25,26^ The freeze-drying process for 5-FU@β-CD/Alg/CEM nanocomposites was optimized using the CHRIST LCG LYO chamber, involving pre-freezing at −80 °C for 12 hours, primary drying at −25 °C under <0.2 mbar vacuum for 30 hours, and secondary drying at +20 °C for 6 hours, totaling approximately 48 hours, to preserve nanocomposite morphology, drug stability, and reconstitution characteristics. A schematic representation of the isolation of mucilage and preparation of nanocomposites is shown in Fig. 1.

## 3. Characterization of 5-FU@ β-CD/Alg/CEM nanocomposites

### 3.1. Fourier transform infrared spectroscopy

FTIR spectroscopic analysis was used to examine the interaction between the drug, the polymers used, and the formulated nanocomposites. A solid sample was analyzed using the KBr method, and the percent of transmission was recorded with a precision range of 500 to 4000 cm^−1^*via* IR Prestige-21, Shimadzu Germany.^27^

### 3.2. Differential scanning calorimetry and thermogravimetry analysis

The sample's thermal stability was assessed (SDT Q600 V20.9 Build 20). Specimens underwent examination in an environment where the temperature was gradually increased from 30 °C to 600 °C at a rate of 10 °C per minute while maintaining an oxygen flow rate of 20 mL min^−1^.^28^

### 3.3. X-ray diffraction

X-ray diffraction (XRD) was employed to examine the fingerprints of the crystal configuration. Diffraction patterns in an X-ray diffractometer vary depending on the crystallographic phase. After placing the powdered sample on a sample holder, it was illuminated with X-rays at a specific wavelength, and the intensity of the reflected rays was recorded. The Powder XRD analysis was performed using a Jeol JDX-3532 diffractometer system, using monochromatic X-rays and scanning an angular range of 5° to 70° (2*θ*).^29^ The Crystallinity Index (CI) was calculated using the Peak Deconvolution method and the following formula.^30^1
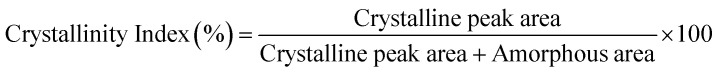


### 3.4. Drug content and %yield

The drug content was assessed using a centrifugation method. The suspension was centrifuged at 15 000 rpm for 40 minutes at 25 °C to separate the free drug. The free drug supernatant was collected and filtered using Whatman filter paper. The absorbance of the filtered solution was measured at 266 nm using a UV spectrophotometer, and the drug content percentage was calculated accordingly.^31^2
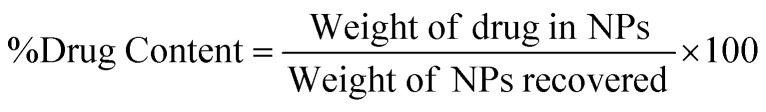
3
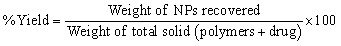


### 3.5. Zeta size and zeta potential analysis

The nanocomposites' electrostatic stability and surface charge were analyzed using zeta potential measurements, while particle size variations in the dispersion solution were evaluated with dynamic light scattering (DLS). Measurements were performed using a Malvern Zetasizer ZS-90 (Malvern Instruments Ltd, U.K). The nanocomposites were dissolved in deionized water at a concentration of 0.5 mg mL^−1^. DLS was performed at a backscattering angle of 173°, and zeta potential measurements were recorded at 25 °C with an applied voltage of 3.3 V.^32^

### 3.6. Scanning electron microscopy and energy dispersive X-ray

The primary focused electromagnetic field was utilized to scan the surface of a specimen (Carl Zeiss, EVO43, SEM, Germany). The specimen's electrons and atoms collaborate to produce a variety of impulses that reveal information regarding the substance of the specimen and surface characteristics. The imaging process associated the signal amplitude with the electron beam's orientation as it scanned in a raster pattern. The Jeol JSM-IT100 microscope, operating at 5 kV, examined the sample at multiple resolutions. Energy-dispersive X-ray spectroscopy (EDS) was used to characterize the sample's chemical and elemental composition.^33^ This technique exploits the unique atomic structures of elements, which generate distinct peaks in their electromagnetic emission spectra, facilitating accurate identification.

### *3.7*. *In vitro* release profile of 5-fluorouracil

The release dynamics of 5-FU@β-CD/Alg/CEM nanocomposites (3 mg) were evaluated by sealing the material within a semi-permeable cellulose membrane and immersing it in 900 mL of release media. Two types of media were used: simulated gastric fluid (HCl/KCl solution, pH 1.2) and simulated intestinal fluid (phosphate–buffered saline, pH 7.4). A dissolution tester (basket method, type 1) maintained at 37.5 ± 0.5 °C with 50 rpm stirring was utilized. Sampling was performed at 13 specific intervals over 96 hours (2, 3, 5, 10, 17, 22, 24, 33, 48, 56, 72, 94, and 96 hours), withdrawing 5 mL of medium and replenishing it with fresh solution each time. The absorbance of the released drug was quantified using a UV-visible spectrophotometer at 266 nm, providing insight into the release kinetics of the nanocomposites.^18^ The release profiles were carried out in triplicate and presented as mean ± S.D.

### 3.8. Drug release kinetics

The release of drug 5-FU has been investigated kinetically by applying a variety of computational models, including “zero-order”, “first-order”, “Higuchi”, “Hixson–Crowell”, and “Korsmeyer–Peppas” approaches.^34^

“Zero-order” the rate of drug release is commonly expressed as being devoid of the concentration of the medicine when the medication dissolves using a dosage form that does not disintegrate and releases the medicine intermittently.^35^

4 *Q*_*t*_ = *Q*_0_ + *k*_0_*t*

“First order” first-order kinetics describes a situation in which a reaction's rate obeys a first-order equation and is exactly proportional to the concentration of one of its components.

5 Log *C* = Log *C*_0_ − *k*_1_*t*/2.303

“Higuchi” Higuchi created models in 1961 (Higuchi 1961) and 1963 (Higuchi 1963) to examine the release of medications incorporated into partially solid and solid composites that were either easily soluble in water or poorly soluble. To examine how a planar system with a homogeneous matrix dissolves, the connection is obtained.^36^

6 *Q*_*t*_ = *kt*^0.5^

“Hixson–Crowell” to assess how variations in the circumference and area of the surface of the fragments or tablets affect medication release, Hixson–Crowell realized in 1931 (Hixson and Crowell, 1931) that the conventional area of a particle is directly proportional to its volume's cubic root, and he proposed an expression as.^37^

7 (*W*_0_^1/3^ − *W*_*t*_^1/3^) = *k*_h_*t*

“Korsmeyer–Peppas” diffusion is the primary drug release mechanism, and in 1983, Korsmeyer *et al.* devised a straightforward semi-empiric paradigm that linearly relates medication release to elapsed time (*t*).^38^

8 *Q*_*t*_/*Q*_∞_ = *k*_p_*t*^*n*^

“where *Q*_0_, *Q*_*t*_, and *Q*_∞_ denote the first 5-FU release rates from the time *t*, as well as time, correspondingly”. “The quantities of 5-FU at the time of *t* & subsequently are *C*_0_ and *C*, correspondingly”. “*W*_0_ and *W*_*t*_ are equal^39^”. 5-FU concentrations in nanoparticles at time *t* and at first, accordingly. The zero-order, first-order, Higuchi, Hixon–Crowell, and Korsmeyer–Peppas coefficients are, respectively, *k*_0_, *k*_1_, *k*, *k*_h_, and *k*_p_. The diffusion factor, or exponent *n*, is used to describe the release of medication strategy.^40^

### 3.9. Cytotoxicity assay

Cytotoxicity assay performed on samples containing @β-CD/Alg/CEM, 5-FU, and 5-FU@β-CD/Alg/CEM nanocomposites. With a few adjustments, the SRB test was performed as previously described.^41^ 10 000 cells per well were placed onto 96-well plates, where they were cultivated for 24 hours before receiving treatment for 48 hours with substances at different doses. Following one to three hours in a cold trichloroacetic acid solution (50%,w/v), the cells were rinsed and dyed for 20 minutes with SRB (0.2%,w/v), and water was used as a negative control. The instrument measured the absorption intensity at 492 nm and 620 nm wavelengths. The percentage of growth inhibition (%) was calculated at a 100 µg mL^−1^ concentration using the following equation.9
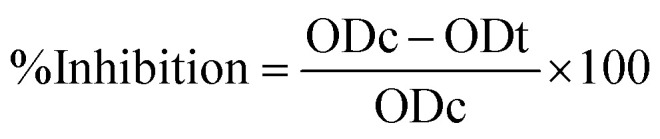
here, ODc and ODt were the optical densities of the negative control and test samples, respectively. A curve-fitting method was used to determine the IC_50_ values graphically.

### 3.10. Statistical analysis

Statistical analysis was performed on the results to determine the statistical significance of the data, and one-way ANOVA, followed by a paired *t*-test, where applicable. The statistical software used was GraphPad Prism 9.5.1. Differences with *p*-values less than 0.001 were considered highly significant. The results are displayed as mean ± SD results from experiments conducted in triplicate.

## 4. Results and discussion

To create nanocomposites of 5-FU@ β-CD/Alg/CEM, an ionotropic gelation process was applied with a few alterations,^18^ combining β-CD monomers and Alg/CaCl_2_/CEM. The ionotropic technique exploited the potential of electrolytes, including polysaccharides, to interact with oppositely charged substances, such as cations. Hydrogels, films, beads, and nanostructures are examples of structured, tangible materials that result from this interaction's sol–gel transformation. With a negative zeta potential, β-CD molecules can interact with alginate to cross-link Ca^2+^ ions, resulting in the formation of insoluble gel-like spheres that form a water-dispersible nanocomposite. The polysaccharide matrix may encapsulate the chemotherapeutic medication 5-fluorouracil (5-FU) by interacting with Ca^2+^ ions and the functional groups of alginate chains and β-CD molecules. Centrifugation may be used to effectively purify and collect the resultant nanocomposite, 5-FU@β-CD/Alg. Notably, a key element in determining the overall effectiveness of the nanocomposite synthesis is the amount. Because drug content has a substantial impact on nanocomposite manufacturing performance, 5-FU@β-CD/Alg/CEM nanocomposites were found to have drug content 87.33 ± 1.75%, while the %yield was found to be 79.06 ± 0.53%.

### 4.1. Characterization of nanocomposites

#### 4.1.1. Fourier transform infrared spectroscopy

**Fig. 2:**
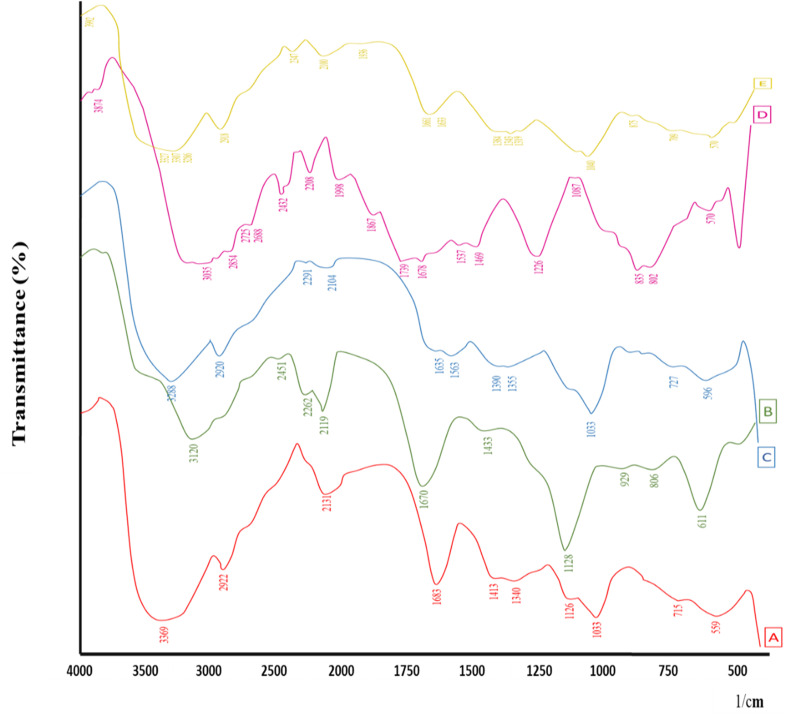
FTIR spectra of (A) β-CD, (B) Alg, (C) CEM, (D) 5-FU, (E) 5-FU@ β-CD/Alg/CEM Nanocomposites.

The β-CD, Alg, CEM, 5-FU, and 5-FU@β-CD/Alg/CEM nanocomposites FTIR spectra are shown in Fig. 2. IR spectra of β-CD (A) showed 3369 cm^−1^ (N–H stretching), 3400–3200^−1^ (O–H stretching vibrations), indicative of wide hydrogen bonding, 2922 cm^−1^ (C–H stretching) alkane, 1639 cm^−1^ (C=C stretching) alkene, and 1340 cm^−1^ (S=O stretching), and a sharp C–O–C stretching peaks in the region of 1150–1030^−1^ corresponding to ether linkages in the glucose units.^42^ IR spectra of CEM showed typical polysaccharide features with a broad O–H stretching around 3430 cm^−1^, aliphatic (C–H stretching) near 2925 cm^−1^, and uronic acid-related C=O stretching between 1730-1600^−1^.^24^ This spectral region (1000–1100 cm^−1^) is typical of C–O and C–OH stretching, highlighting the polysaccharide framework of the mucilage.

IR spectra of pure drug 5-FU (D) represented characteristic peaks at 3034 cm^−1^ (N–H stretching), 2852 cm^−1^ (C–H stretching), 1737 cm^−1^ (C=O stretching), and the band showed a peak of 1224 cm^−1^ (C–F stretching) and (C–N stretching vibrations between 810–750 cm^−1^ confirming the presence of fluorinated and amide functional groups.^42^ The FTIR spectrum of the 5-FU@β-CD/Alg/CEM nanocomposite (E) exhibited several notable peak shifts and modifications, indicating the successful encapsulation of the drug within the tri-polymer matrix. The broad and intensified O–H stretching vibration observed between 3400 and 3200 cm^−1^ in the nanocomposite spectrum indicates robust intermolecular hydrogen bonding among β-CD, Alg, CEM, and 5-FU. A notable shift in the C=O stretching band of 5-FU from 1680 cm^−1^ to approximately 1620 cm^−1^ suggested the presence of hydrogen bonding or an electrostatic interaction between the drug and the carboxylate functionalities of sodium alginate. Additionally, minor shifts in the asymmetric and symmetric COO^−^ stretching bands of alginate further support this interaction. The marked decrease in intensity of the C–F stretching band near 750 cm^−1^ in the composite implies successful encapsulation of 5-FU and potential shielding effects by the polymer matrix. Furthermore, the enhanced overlapping peaks within the 1100–1030 cm^−1^ region correspond to combined C–O–C and C–OH vibrations from all three polysaccharides, confirming the successful formation of a structurally integrated nanocomposite. The major peak positions and shifts of 5-FU and 5-FU@β-CD/Alg/CEM Nanocomposites are compared in Table 1.

**Table 1:** Comparison of major peak positions and shifts of 5-FU and 5-FU@β-CD/Alg/CEM Nanocomposites

Functional group/vibration	5-FU (cm^−1^)	5-FU@β-CD/Alg/CEM nanocomposite (cm^−1^)	Interpretation
N–H stretching	3034	3400–3200 (overlapped, broad)	Strong intermolecular H-bonding with polymers
C–H stretching	2852	2920–2925 (merged)	Retained, minor overlap
C=O stretching	1737–1680	1620 (shifted)	Interaction with Alg carboxylate groups (H-bonding/electrostatic)
C–F stretching	1224, 810–750	750 (reduced intensity)	Shielding due to encapsulation in a polymer matrix
C–N stretching	810–750	Overlapped, less intense	Indicates interaction with polymer network

### 4.2. Thermogravimetric analysis (TGA)

**Fig. 3:**
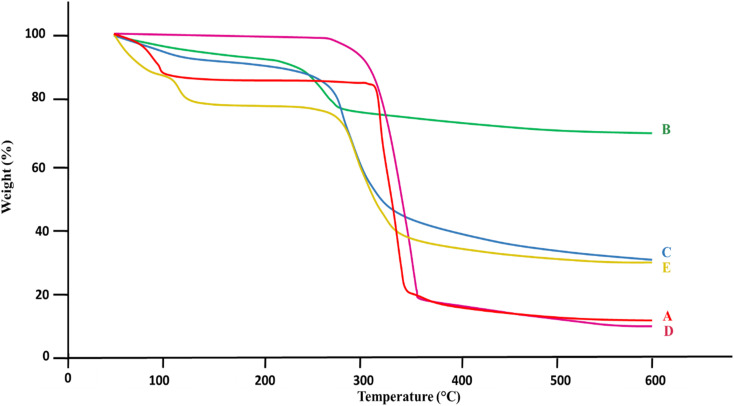
TGA thermograms overlay (A) β-CD, (B) Alg, (C) CEM, (D) 5-FU, (E) 5-FU@β-CD/Alg/CEM Nanocomposites.

Thermal analysis of β-CD, Alg, CEM, 5-FU, and 5-FU@β-CD/Alg/CEM nanocomposites is shown in Fig. 3. The thermal analysis for β-CD (A) revealed two main weight loss phases.^43^ The loss of adsorbed water was probably the cause of the initial small weight loss at 100 °C. Since β-CD's biological composition decomposed at higher temperatures, the substantial decrease in weight at 300 °C signified the breakdown of the compound.^44^ Alginate (B) maintained a weight retention up to 200 °C; beyond that, a notable weight loss. Alginate materials typically exhibited such characteristics, with degradation starting between 200 and 250 °C, probably due to the breakdown of the structural polymer component. At higher temperatures, alginate maintained weight compared to alternative samples, suggesting comparatively superior thermal resistance.^45^ The thermal analysis of CEM(C) presented modest weight loss at first, followed by a substantial reduction in weight at approximately 300 °C. This suggested that CEM degraded identically to β-CD. The reduction in weight profile showed that the framework of CEM degraded at higher temperatures and that volatile substances were released at lower temperatures.^46^

Substantial weight loss was shown in the 5-FU (D) thermogram beginning at 250 °C, with a major degradation onset at approximately 265 °C – 270 °C and steep declines that completed around 330 °C. These findings showed that 5-FU degraded rapidly at lower temperatures because of its limited thermal stability. 5-FU, being a small-molecule drug, exhibited rapid weight loss and showed poor thermal endurance.^44^ In contrast, the 5-FU@β-CD/Alg/CEM nanocomposites (E) showed a delayed onset of degradation, around 280 – 290 °C, with a broader and more gradual decomposition profile. The major weight loss was less steep compared to pure 5-FU, and residual mass at higher temperatures was greater, indicating improved thermal stability. This suggested that 5-FU was thermally protected by encapsulation in β-CD/Alg/CEM, increasing the composite's stability, and this could prove useful in applications that call for regulated release at high temperatures.

### 4.3. Differential scanning calorimetry (DSC)

**Fig. 4:**
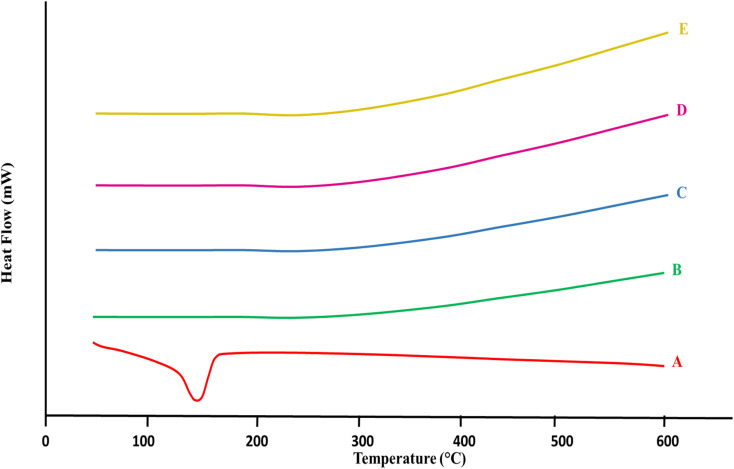
DSC thermograms overlay (A) β-CD, (B) Alg, (C) CEM, (D) 5-FU, (E) 5-FU@β-CD/Alg/CEM nanocomposites.

In every specimen of Fig. 4(A–E), the given DSC (Differential Scanning Calorimetry) thermogram overlaid the specimen's thermal pattern as an indicator of temperatures. Between 100 to 150 °C, the β-CD (A) thermogram displayed an endothermic peak that probably represented the expulsion of accumulated water or moisture because of crystallization. The thermodynamic flow became more stable following that peak. The absence of any other prominent endothermic peaks suggested that, under DSC analytical circumstances, β-CD did not melt or decompose much in this range of temperatures.^47^ When the temperature increased, alginate (B) showed a steady flow of heat patterns with a slight elevated at the start, indicating a slow uptake of heat with no noticeable thermodynamic changes. Alginate exhibited strong thermal stability and no abrupt phase changes within the measured range, according to this consistent trend.^48^

The thermal image of CEM (C) displayed a stable base with a slow rise in the flow of heat. Like alginate, it showed no notable endothermic or exothermic spikes in this temperature range, suggesting that there were no major changes such as melting or breakdown.^49^ 5-FU (D) represented a clear endothermic peak around 160– 180 °C, demonstrating its melting point and onset of degradation. This peak was quite sharp because 5-FU is a small, crystalline drug molecule.^47^ In contrast, 5-FU@β-CD/Alg/CEM nanocomposites (E) showed no prominent peak in this area, showing the absence of crystalline property of 5-FU, or it might be converted to an amorphous form within the formulated polymer matrix nanocomposites. Hence, the nanocomposites curve was shifted beyond 200 °C, indicating improved thermal stability of 5-FU when embedded in β-CD/Alg/CEM matrix, attributed to drug–polymer interaction that reduced the mobility of 5-FU and delayed decomposition. A summary of the TGA and DSC findings for all samples is presented in Table 2.

**Table 2:** Summary of TGA and DSC findings of β-CD, Alg, CEM, 5-FU, and 5-FU@β-CD/Alg/CEM nanocomposites

Sample	TGA onset of degradation (°C)	Major weight loss/Peak (°C)	Residual Mass (high T)	DSC key features	Remarks/Interpretation
β-CD	−100 (water loss)	−300 (major breakdown)	Low	Endothermic peak 100–150 (moisture loss)	Decomposes at higher *T* due to biological structure
Alg	−200	200–250 (polymer degradation)	Moderate-high	Stable heat flow, no sharp peaks	Superior thermal resistance among polymers
CEM	−100 (minor loss)	−300 (framework degradation)	Low-moderate	Stable baseline, no sharp peaks	Similar degradation pattern to β-CD
5-FU	−250	265–270 (sharp degradation) → complete −330	Very low	Sharp endothermic peak 160–180 (melting/degradation)	Poor thermal stability, crystalline small molecule
Nanocomposite (5-FU@β-CD/Alg/CEM)	−280–290 (delayed onset)	Broader, gradual decomposition >300	Higher residual mass	No sharp melting peak; curve shifted beyond 200 → amorphous form	Improved thermal stability; encapsulation protects 5-FU

### 4.4. X-ray diffraction

**Fig. 5:**
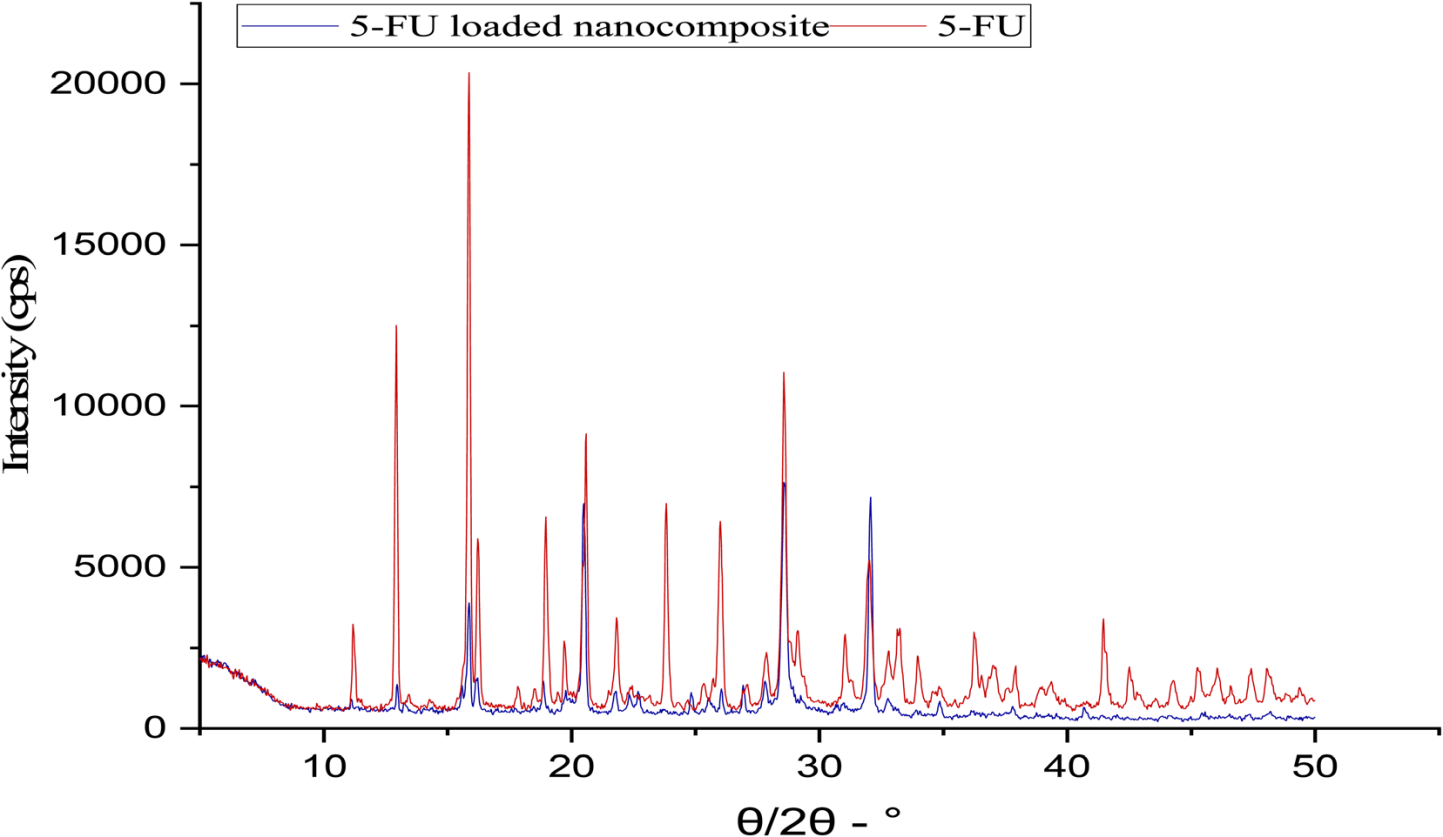
XRD of 5-FU and 5-FU@β-CD/Alg/CEM nanocomposites.

XRD spectra of the pure 5-FU and 5-FU@β-CD/Alg/CEM nanocomposites were contrasted as shown in Fig. 5. There were distinct, intense peaks in the 5-FU sample at several locations, particularly at 2*θ* values of 12.1°, 16.0°, 18.2°, 21.2°, 24.5°, 28.56°, and 31.6°, revealing the compound's crystal-like morphology in the pure 5-FU sample.^50^ XRD of 5-FU@β-CD/Alg/CEM nanocomposites revealed that the 12.1° sharp peak was very weak, the 16.0° sharp peak widen, 18.2° very intense peak broadened showing a significant reduction in the crystallinity of 5-FU; 21.2° sharp peak has disappeared, 24.5° strong peak became slightly broader, 28.56° moderate peak converted to broader hump around 29°, 31.6° moderate peak converted to very weak hump reflecting a reduced crystallinity of 5-FU. The crystallinity index of pure 5-FU was 88.48%. In contrast, it decreased to 58.96% in the 5-FU@β-CD/Alg/CEM, indicating a reduction in crystallinity due to molecular dispersion of the drug within the polymer matrix.

### 4.5. Zeta potential and zeta size analysis

**Fig. 6:**
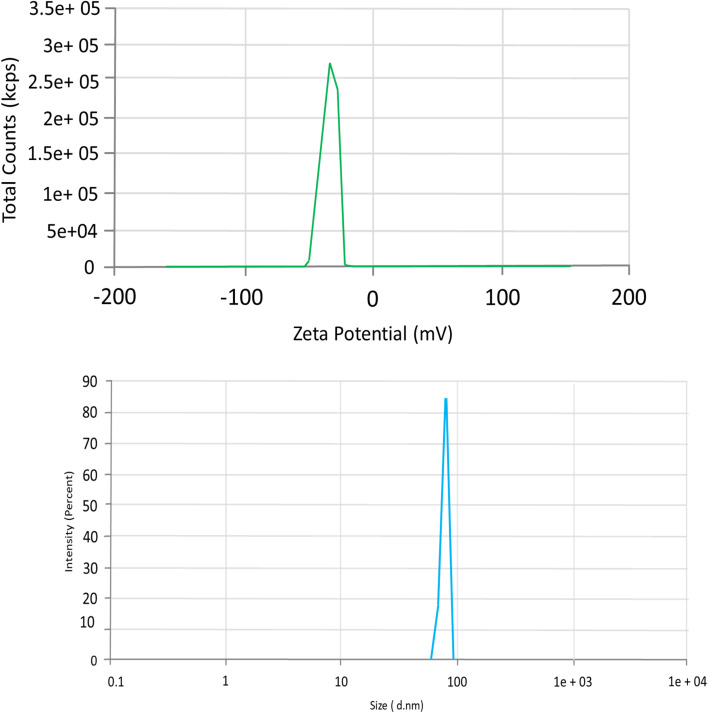
(A) Zeta-potential (mV) of 5-FU@β-CD/Alg/CEM nanocomposites. (B) Z-average hydrodynamic diameter (nm) of 5-FU@β-CD/Alg/CEM nanocomposites measured by DLS.

The zeta potential of the nanocomposites was measured to reveal their surface charges, which were −27.1 mV (Fig. 6A), indicating that the nanoparticles increased stability. A minimum of ±30 mV is required for electrostatically stabilized suspensions, and a minimum of ±20 mV is desirable in the case of electrostatic and steric stabilization. β-CD and CEM primarily contributed to steric stabilization due to their bulky molecular structures and surface-bound polysaccharide chains, while Alg mainly provides electrostatic stabilization because of its negatively charged carboxylate groups.^51,52^ This result implies that stability over an extended period might be achieved by preventing particle agglomeration and aggregation through the presence of repellent effects. The variation in particle size of tiny particles distributed in liquid or suspension form can be ascertained effectively and analytically with DLS. Fig. 6B illustrates the average size ranges of the produced 5-FU@β-CD/Alg/CEM nanocomposites, indicating the intensity. The gathered particles were shown to be a polydisperse mixture through laser spectrum testing. The average particle size was determined to be 80–100 nm with a PDI of 0.611. Although the nanocomposites exhibited a negative zeta potential and steric stabilization from β-cyclodextrin and cress seed mucilage, the relatively high PDI value (0.611) indicated a broad particle size distribution. This variation cannot be attributed to steric hindrance alone. The broader distribution likely arose from several formulation-related factors, including the intrinsic heterogeneity of the natural mucilage, differences in polymer chain length, and variability in crosslinking density during ionotropic gelation. Additionally, the complex interpolymer interactions among β-CD, alginate, and mucilage may lead to the formation of particles of multiple size populations. Despite this broader PDI, the system remained physically stable without noticeable aggregation, suggesting that the combined steric and electrostatic contributions were sufficient to maintain dispersion stability.^50^

### 4.6. SEM and EDX

**Fig. 7:**
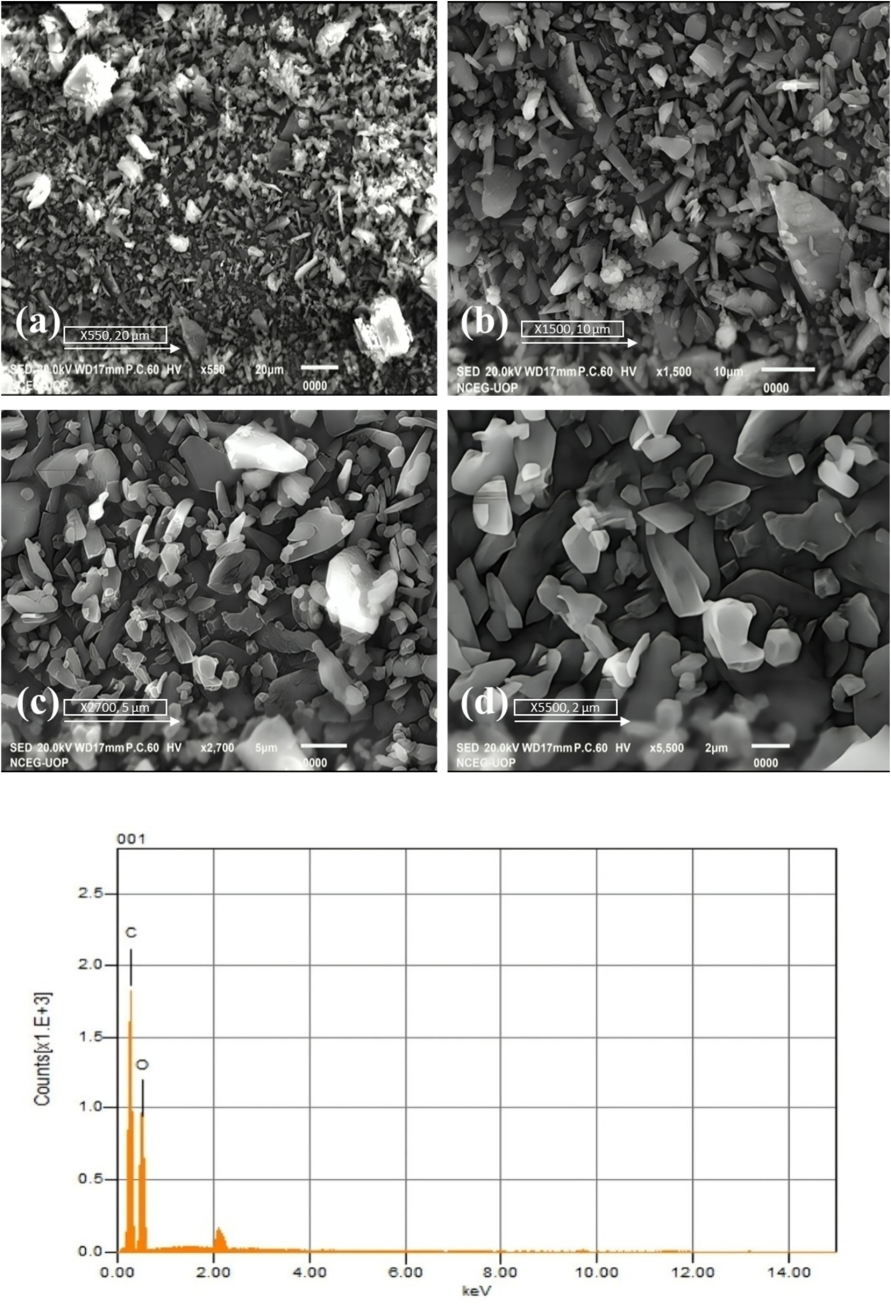
(A) SEM images of 5-FU@β-CD/Alg/CEM nanocomposites (a) 20 µm, (b) 10 µm, (c) 5 µm, (d) 2 µm. (B) EDX spectrum of 5-FU@β-CD/Alg/CEM nanocomposites showing elemental composition.

Fig. 7A displayed a surface morphology of 5-FU@β-CD/Alg/CEM Nanocomposites at various magnifications that was uneven and irregular, which was typical of composite components. The porous and rough surface might be embedded in a matrix made of CEM, β-CD, and Alg, which would serve as a bonding framework or carriers for 5-FU. Aggregation of small fragments having a small gap was seen in several regions at greater intensities, especially in the bottom right picture, which may be a sign of clustering. The existence of simultaneous porosity and dense portions may indicate a complicated and multi-phase arrangement in the nanocomposite that can affect the drug release pattern.^33^ The detected appearance of roughness and porousness may help with drug incorporation and release because a coarse perforated area offers more spots for drug attachment and potentially controls the release of the drug. Although the arrangement of particles appears to be rather equal over the entire surface, certain bigger, identifiable nanoparticles, likely alginate or β-CD encapsulation shells, are apparent among the smaller particles, maybe because of the filamentous character of CEM.^53^

SEM was employed in collaboration with EDX. A beam of electrons containing a voltage of 10–20 keV, which causes the material to release X-rays imaging, strikes the electrically conducting specimen. The substance being studied determined the power of the X-rays released. EDX was widely utilized in compositional evaluation and spectrum analysis to confirm the composition and dispersion of nanomaterials. The findings of an EDX investigation on nanocomposites containing 5-FU@β-CD/Alg/CEM were displayed in Fig. 7B. According to EDX, the produced nanocomposites contain elements such as C and a noticeable peak that is located adjacent to the low-energy band (around 0.3 keV), represented by O. Carbon. Given that carbon was a major constituent of organic compounds, including β-CD, Alg, CEM, and 5-FU, this seemed comprehensible. Oxygen (O): because both the polysaccharides (Alg & CE) and β-CD constituents in the nanocomposite are oxygen-rich, the second prominent peak, signifying oxygen, was seen close to 0.5 keV.^54^

The absence of notable peaks for other elements in the spectrum suggested that the substance was mostly made up of carbon and oxygen. This might indicate that a composite consisting primarily of organic components was successfully formed under the anticipated constitution. In contrast to oxygen, carbon had a greater count intensity (*y*-axis), indicating that it was more prevalent in the specimen. When the carbon framework predominated in organic-based nanocomposites, this took place normally. According to the mixture of 5-FU, β-CD, Alg, and CEM, EDX testing confirmed that carbon and oxygen were the main constituents in the nanocomposite. With no discernible contaminants by inorganic compounds, this compositional profile indicated that the nanocomposite formulation might have effectively contained 5-FU inside a β-CD/Alg/CEM matrix, as shown in Fig. 7A and B.

### 4.7. *In vitro* drug release of 5-FU@β-CD/Alg/CEM nanocomposites under pH-responsive conditions

**Fig. 8:**
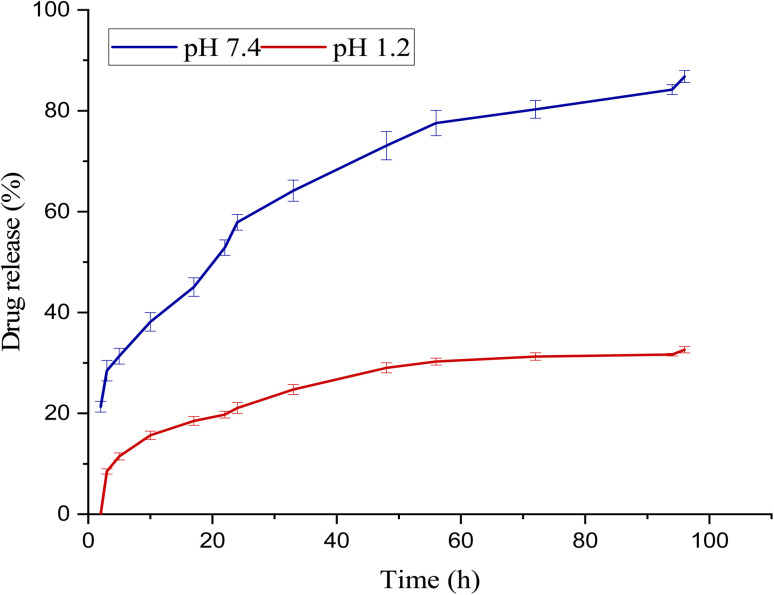
*In vitro* release profile of 5-FU@β-CD/Alg/CEM nanocomposites at pH 1.2 and pH 7.4 (*n* = 3/mean ± S.D). Bars denote a highly significant difference between formulations (*p* < 0.001).

The *in vitro* release kinetics of 5-FU from the β-CD/Alg/CEM nanocomposites were investigated in two different simulated physiological environments: acidic medium (pH 1.2) and phosphate-buffered saline (pH 7.4), representing gastric and intestinal conditions, respectively, as shown in Fig. 8. Under intestinal conditions (pH 7.4), 5-FU was released more significantly and continuously throughout the period; the rate of release rose quickly in the first few hours and then gradually, reaching around 85% over 96 hours. In contrast, in the acidic medium (pH 1.2), the release rate was much lower and only reached around 25% at the end of the observed period, indicating that 5-FU was either less soluble in acidic environments, which lowered the release rate. This pH-responsive release behavior can be attributed to the ionization state and solubility profile of the polymeric matrix components. At pH 7.4, the alginate and mucilage-based network becomes more hydrated and swells due to increased ionization of carboxylic groups, facilitating matrix erosion and enhanced diffusion of 5-FU. In contrast, at pH 1.2, the network remains more compact and protonated, restricting water uptake and drug mobility, thereby minimizing premature release in the gastric environment. β-CD/Alg/CEM nanocomposite demonstrated a markedly sustained and controlled release pattern in both acidic and physiological media. This slower and extended-release profile indicated the structural advantage of the β-CD cavity and the Alg/CEM matrix in retaining the drug, reducing initial burst release, and providing prolonged drug availability. These findings suggested the potential of our nanocomposite to overcome the rapid release and short systemic residence time associated with free 5-FU.^55^

### 4.8. Drug release kinetics

The five main mathematical models each included a release kinetics model for the 5-FU medication in different media. The Korsmeyer–Peppas model may be the best model for explaining the release kinetics in this dataset. Subsequently, it fits the data the best with the greatest *R*^2^ values of 0.9021 for pH 1.2 and 0.9903 for pH 7.4, and MSC values as shown in Table 3. The Korsmeyer–Peppas model was most appropriate due to its relevance in describing diffusion and polymer relaxation behavior. The *n* values (0.378 at pH 1.2 and 0.343 at pH 7.4) suggest a Fickian diffusion mechanism (*n* < 0.45), a common mechanism in polymer-based controlled release formulations.^56^ This implies that drug release was controlled predominantly by molecular diffusion through the hydrated polymer matrix rather than by swelling or erosion processes. In a physiological context, this finding is particularly relevant to gastrointestinal transit. In the acidic gastric environment (pH 1.2), the reduced swelling and tighter polymer network restricted diffusion, resulting in slower drug release. In contrast, under intestinal pH conditions (pH 6.8–7.4), increased ionization of alginate and enhanced polymer relaxation facilitated higher water uptake and matrix swelling, thereby accelerating diffusion-driven drug release. Since residence time in the small intestine (3–4 h) is typically longer than in the stomach (1–2 h), the observed pH-responsive, diffusion-controlled release profile suggested that the nanocomposite system could preferentially release the drug in the intestine, improving bioavailability and reducing gastric side effects.

**Table 3:** Kinetics of drug release at pH 1.2 & pH 7.4

Model	Parameter	pH 1.2	pH 7.4
Zero-order	*R* ^2^	0.139	0.1370
	MSC	0.4991	0.2823
First order	*R* ^2^	0.3394	0.7791
	MSC	0.2351	1.3561
Hixson–Crowell	*R* ^2^	0.2767	0.6615
	MSC	0.3258	0.9294
Higuchi	*R* ^2^	0.8484	0.8723
	MSC	1.2370	1.9044
Korsmeyer–Peppas	*R* ^2^	0.9021	0.9903
	MSC	1.5204	2.3550
	*n*	0.378	0.3430

### 4.9. Cytotoxic study of 5-FU@β-CD/Alg/CEM nanocomposites

The cytotoxic characteristics of 5-FU, @β-CD/Alg/CEM, 5-FU@β-CD/Alg/CEM, and release solutions at numerous pH levels after 48 hours against a breast cancer cell line (MCF-7 cell lines were provided by the University of Lahore's cell BioBank (IMBB/CRiMM), and a healthy cell line (the fibroblast) are demonstrated. MCF-7 cells have a modest inhibitory impact from the β-CD/Alg/CEM complex, with an inhibition of 32.46 ± 2.99%. According to this, the β-CD/Alg/CEM complex, by itself, may be able to inhibit the proliferation of breast cancer cells to some extent; however, its effectiveness was far less compared to any of the other therapies examined. With a fibroblast inhibition rate of 1.17 ± 3.99%, it was quite low. This suggested that the complex targeted cancer cells more than healthy ones, exhibiting selectivity that may be useful for therapeutic applications and displaying negligible cytotoxicity against healthy fibroblast cells. With an IC_50_ value of >100 µg mL^−1^ against MCF-7 cells, β-CD/Alg/CEM appears to have a considerable cytotoxic effect, resulting in 50% mortality at this dose. IC_50_ on Fibroblast Cells: β-CD/Alg/CEM exhibits little toxicity to fibroblast cells, with an IC_50_ value greater than 100 µg mL^−1^. The fact that this compound is more deadly to cancer cells than healthy ones lend greater credence to its selective activity.

5-FU demonstrated strong cytotoxicity against MCF-7 cells, achieving 82.31 ± 3.55% inhibition. As anticipated, for this commonly used anticancer medication, this substantial inhibition validated the high-killing action of 5-FU on breast cancer cells. With an inhibitory percentage of 49.26 ± 4.82% against fibroblast cells, 5-FU also exhibited strong cytotoxicity toward normal cells, which might have negative implications in therapeutic contexts. 5-FU showed strong cytotoxicity against MCF-7 cells, inducing 50% cell death at low concentrations (IC50 = 3.25 ± 1.37 µg mL^−1^), highlighting its potent anticancer activity. Although fibroblasts exhibited considerable suppression, the IC_50_ result of >100 µg mL^−1^ indicated that the quantities examined do not surpass the fatal limit for regular cells.

The 5-FU@β-CD/Alg/CEM nanocomposite exhibited 90.69 ± 3.95% inhibition of MCF-7 cells, which was significantly higher than that of free 5-FU, indicating enhanced anticancer activity. The encapsulation of 5-FU in the β-CD/Alg/CEM nanocomposite might control the drug's release, resulting in sustained, but slightly reduced, activity compared to free 5-FU. For fibroblasts, the inhibition is 19.35 ± 2.31%, considerably lower than the inhibition caused by free 5-FU. This suggests that the nanocomposite formulation reduces the cytotoxicity of 5-FU on normal cells, potentially lowering side effects. IC_50_ on Fibroblast Cells is >100 µg mL^−1^, indicating that the nanocomposite does not reach the lethal dose for either cell line within the tested concentration range. This result showed that the nanocomposite facilitated improved internalization by cancer cells (MCF-7), increasing intracellular drug concentration and efficacy while potentially reducing exposure to healthy cells. By controlling the release and improving localization, the nanocomposite minimized the immediate systemic availability of 5-FU, thereby reducing off-target effects. Cytotoxicity profiles of dummy (β-CD/Alg/CEM nanocomposites), standard (5-FU), and prepared formulation (5-FU@β-CD/Alg/CEM nanocomposites) are shown in Table 4.

**Table 4:** Cytotoxicity of β-CD/Alg/CEM nanocomposites, 5 FU, and 5-FU@β-CD/Alg/CEM nanocomposites

No.	Samples	Inhibition^a^		IC_50_ (µg mL^−1^)	
		MCF-7	Fibroblast	MCF-7	Fibroblast
1	β-CD/Alg/CEM	32.46 ± 2.99a	1.17 ± 3.99a	>100d	>100
2	5-FU	82.31 ± 3.55c	49.26 ± 4.82c	3.25 ± 1.37a	>100
3	5-FU@β-CD/Alg/CEM nanocomposites	90.69 ± 3.95b	19.35 ± 2.31b	20.85 ± 1.31c	>100

a The samples were tested at a concentration of 100 µg mL^−1^. Statistically significant differences (*p* < 0.05) between groups are denoted by different letters (a–d). Each value represents the mean of three independent biological replicates, with each biological replicate measured in triplicate (technical replicates).

The findings demonstrate each sample's unique cytotoxic profiles: by itself, β-CD/Alg/CEM exhibits minimal cytotoxicity toward fibroblasts and minor selectivity toward cancer cells. By controlling the release and improving localization, the nanocomposite minimized the immediate systemic availability of free 5-FU, thereby reducing off-target effects. Therefore, this encapsulation technique may be a good way to target cancer cells while minimizing negative effects on healthy cells. According to these results, the 5-FU@β-CD/Alg/CEM nanocomposite may be the most attractive option for future research in cancer treatment since it may combine increased safety and efficacy.

## 5. Conclusion

This study successfully developed and characterized 5-FU-loaded β-CD/Alg/CEM nanocomposite through a promising feature of targeted cancer therapy. The zeta potential analysis confirmed a stable colloidal solution, displaying negative values of −27.1 mV. The release profile of 5-FU was significantly lower in a highly acidic environment (pH 1.2) compared to a neutral environment (pH 7.4), demonstrating the preparation potential for controlled drug release while minimizing drug degradation in the gastric environment. Cytotoxicity tests confirmed effective and selective inhibition of MCF-7 cancer cells with limited toxicity toward normal cells. However, the study is limited to *in vitro* findings; long-term stability and *in vivo* therapeutic performance were not evaluated. Future work should address these limitations through *in vivo* studies and formulation optimization to support the potential clinical translation of this tripolymer-based nanocarrier system.

## Author contributions

Maham Anoosh: conceptualization, methodology, formal analysis, writing – original draft, data curation, investigation; Shazia Akram Ghumman: conceptualization, supervision, project administration, investigation; Huma Hameed: methodology, validation, visualization, writing – review & editing; Shazia Noureen: data curation, visualization; review & editing; Rizwana Kausar: formal analysis, writing – review & editing; Ali Irfan.; formal analysis, funding acquisition, visualization, writing – review & editing; Maged Ali A. Alrobesh: data curation; funding acquisition, investigation, writing – review & editing; Bakar Bin Khatab Abbasi, formal analysis, investigation, writing, review & editing; Pervaiz Akhtar Shah: software analysis, data curation, visualization; writing, review & editing; Maria Rana: validation, resources, data curation, Yousef A. Bin Jardan: funding acquisition, formal analysis, visualization; project administration, writing – review & editing. All authors have read and agreed to the published version of the manuscript.

## Conflicts of interest

The authors have no conflicts of interest to declare.

## Data Availability

All the data of this study are contained in the manuscript. For any additional data or information needed regarding this research, please contact the corresponding author, Shazia Akram Ghumman.
